# Current Understanding of PCSK9 and Its Relevance to Cancer Prognosis and Immune Therapy: A Review

**DOI:** 10.30699/IJP.2023.1999459.3093

**Published:** 2023-12-29

**Authors:** Morteza Hassandokht Mashhadi, Fahime Taheri, Sadaf Irani, Arshiya Mesbah Mousavi, Ali Mehri, Hossein Javid

**Affiliations:** 1 *Department of Medical Laboratory Sciences, Varastegan Institute for Medical Sciences, Mashhad, Iran*; 2 *Endoscopic and Minimally Invasive Surgery Research Center, Ghaem Hospital, Mashhad University of Medical Sciences, Mashhad, Iran*; 3 *Department of Clinical Biochemistry, Faculty of Medicine, Mashhad University of * *‎* *Medical Sciences, Mashhad, Iran*

**Keywords:** Cancer, Immunotherapy, Immune checkpoint, PCSK9

## Abstract

The effectiveness of immunotherapy for most cancer patients remains low, with approximately 10–30% of those treated surviving. Thus, much effort is being put into finding new ways to improve immune checkpoint therapy. Our review concludes that inhibition of proprotein convertase subtilisin/Kexin type 9 (PCSK9), which plays a critical role in regulating cholesterol metabolism, can cause movement of T cells toward tumors, with increased sensitivity to immune checkpoint therapies.

We searched PubMed, NCBI, Scopus, and Google Scholar for the published articles without limitations on publication dates. We used the following terms: “PCSK9”, “Cancer”, “Immune Checkpoint”, and “Cancer Prognosis” in the title and/or abstract. Our search initially revealed 600 records on the subject and stored them in the used databases under EndNote X8 management software. A total of 161 articles were selected and through a careful review, 76 were included in our research.

We concluded that PCSK9 reduces the number of LDL receptors (LDL-R) on the cell surface, which is linked to its ability to regulate cholesterol levels in the body. Also, we discuss how suppressing PCSK9 leads to the MHC-1 accumulation on the surface of cancer cells, which results in T lymphocyte invasion. Finally, we believe that inhibiting PCSK9 may be an effective strategy for improving cancer immunotherapy.

## Introduction

Proprotein convertases (PCs) are serine proteases that convert various growth factors, cell surface glycoproteins, receptors, and metalloproteinases into active forms (1). PCSK9 is an enzyme and the nin^th^ member of proprotein convertases that activates other proteins and also plays a vital role in regulating low-density lipoprotein cholesterol (LDL-C) levels because of its ability to adjust the hepatic expression of LDL-R (2). PCSK9 is produced in many organs, including kidneys, intestine, endocrine pancreas, and brain, but most significantly in the liver (3). Recent studies have revealed its presence in cerebrospinal fluid (CSF) and in the atherosclerosis plaque (4).

**Fig. 1:**
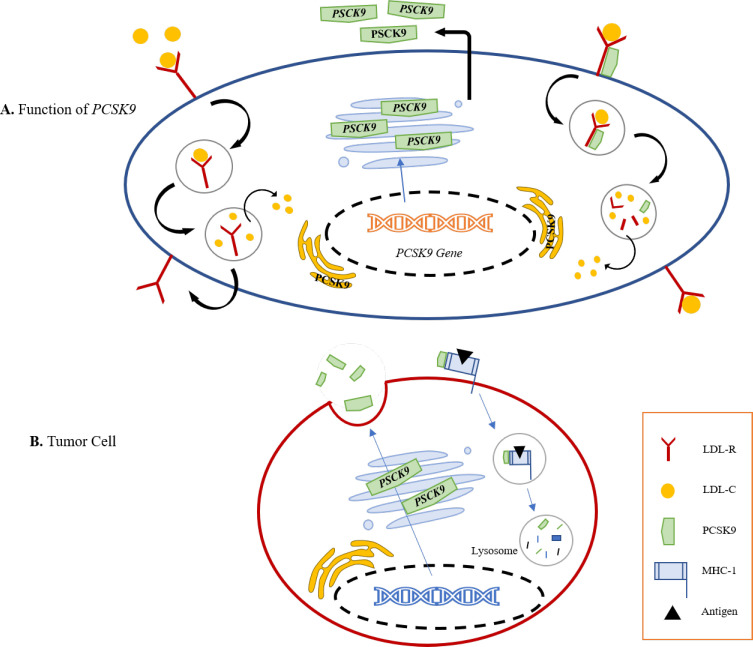
**A.** Role of PCSK9 in increasing cholesterol availability for cancer cells (24). **B.** In tumor cells, upon binding to MHC I, PCSK9 mediates degradation via the endosomal/lysosomal pathway, preventing its recycling to the surface

The inhibition of PCSK9 may be a promising treatment option to reduce LDL-C levels, though the cost of PCSK9 inhibitors may make their wide use difficult (5) (Figure 1).

In this study, we demonstrate that inhibition of PCSK9 might enhance the anti-cancer effects of immune checkpoint inhibitors (ICIs) (6). Therefore, a better understanding of PCSK9's role in the cancerous pathways is extremely important (7). Since this marker has a significant role in different parts of body, it has been used as a pharmacological target in the past few years (8-10). 

Immune checkpoints are potent antioxidant regulators that control cellular tolerance and prevent tumors. Immune checkpoints assist the immune system in response to infections and cancer, which may protect tissues from damage (11-13). The concept that the immune system might restrict tumor development and cancer, dates back to 1893, when William Cooley used live bacteria as an immunological stimulant to treat cancer patients (2). This natural biological cancer-prevention mechanism has already been identified and has been activated by the essential immune inspection chemicals in cytotoxic T cells (14). The CTLA-4, PD-1, and PD-L1 are the most widely studied inhibitory pathways, and by blocking them, we can activate the immune system to attack tumors (15-17). Ipilimumab [CTLA-4 monoclonal antibody (18)] was approved by the Food and Drug Administration (FDA) as the first ICI in 2011 (19). Checkpoint therapy for cancer includes strategies that enhance the immune system response against tumor cells by targeting these regulatory pathways (20).

On the other hand, we have ICIs that have emerged as one of the most promising types of immunotherapies on the horizon in recent years. ICIs are the latest breakthrough in oncology, providing a new treatment model for advanced solid tumors (21). This shifted the companies’ focus away from developing cancer-fighting therapies toward screening inhibitors, which aim to kill tumor cells by removing obstructive signals that block anti-tumor T cell responses (22). In summary, we believe blocking PCSK9 would be a promising approach for improving cancer immune checkpoint therapy (23).

## Methods

We searched PubMed, NCBI, Scopus, and Google Scholar for the published articles without limitations on publication dates. The first search used the following terms: “PCSK9”, “Cancer”, “Immune Checkpoint” and “Cancer Prognosis” in the title and/or abstract. Our search initially revealed 600 records in used databases managed by the EndNote X8 software and we chose around 200 articles that were proper for our research. A total of 39 duplicate references were removed. The full texts of the remaining 161 articles were carefully reviewed and 76 of them were included in our research.

**1. PCSK9 in Carcinoma**

The discovery that PCSK9 interacts with LDL-R marked a significant advancement because it allowed the development of effective therapeutic strategies for cancer and other diseases (25). This section discusses PCSK9's potential as a biomarker for specific types of cancers.

**1.1. PCSK9 and Hepatocellular Carcinoma**

**Fig. 2:**
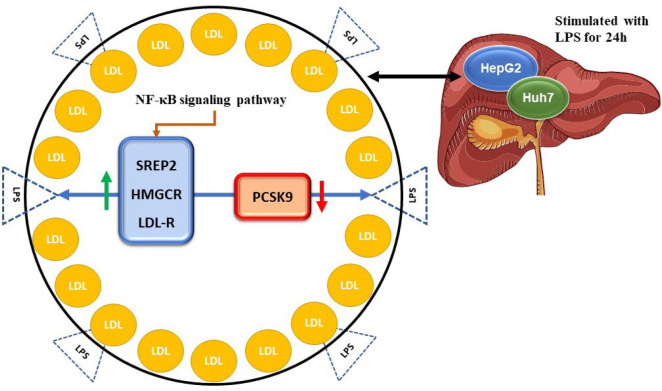
The schematic representation effect of LPS on PCSK9 in HCC (32, 36, 37).

Hepatocellular carcinoma (HCC) is the fifth malignant neoplasm and the third most prominent cause of cancer death (26). The link between abnormal blood lipid levels and HCC has been established in clinical studies by He* et al.*, (1, 27); They discovered that PCSK9 reduces HCC cell growth, cell cycle, and apoptosis in HepG2 cell line (a cell culture created from a single cell and contains cells with a consistent genetic makeup) by interacting with glutathione S-transferase p1 (GSTP1) and the c-Jun N-terminal kinase (JNK) signaling pathway (Up-regulated way) (28, 29). Still, the cell cycle study didn't find any G2/M phase arrest when PCSK9 was overexpressed or down-regulated (29). This means that PCSK9 doesn't have a big effect on how HCC cells divide (30). However, it has an impact on apoptosis with inhibitory effect (31). PCSK9 hinders the use of LDL and triglycerides by destroying LDLR (32). Fatty acid synthase (FASN) is expressed more often when PCSK9 is present (26, 30). It plays a crucial role in the synthesis of fatty acids from the beginning (29, 33). It also has a significant impact on the apoptosis of a variety of tumor types (34). Nevertheless, PCSK9 expression is unaffected by FASN blocking, which may lessen the anti-apoptotic impact (26). These results imply that FASN is downstream of PCSK9 in the apoptosis regulation mechanism (35). This research showed that FASN-mediated anti-apoptosis was crucial to the formation of HCC and that PCSK9 facilitated this proliferation (26, 30, 35). Nowadays, it has been found that using lipopolysaccharide (LPS) causes PCSK9 expression to be down-regulated while the expressions of SREBP2, HMGCR, and LDL-R are up-regulated (Figure 2) (32). In malignancies, PCSK9 expression was associated with poor outcomes in patients with HCC.

LPS is used for 24 hours to activate HepG2 and Huh7 cell lines as human HCC cells and significantly enhances intracellular cholesterol levels by increasing the expression of SREBP2, HMGCR, and LDL-R while down-regulating the expression of PCSK9 (Down-regulated way). Surprisingly, these results relied on the nuclear factor kappa-light-chain-enhancer of activated B cells (NF-kB) signaling pathway. 

**1.2. PCSK9 and Lung Cancer**

Non-small cell lung cancer (NSCLC) is the most common lung cancer type, with more than 80% of all occurrences (38). A study has shown that PCSK9 levels in lung tumor samples are considerably lower than in normal surrounding tissue (32). By targeting adenocarcinoma in NSCLC therapy, human alveolar basal epithelial cells (A549, a cell line of human lung adenocarcinoma) were transfected with PCSK9 siRNA, and it was discovered that PCSK9 siRNA could inhibit proliferation and increase apoptosis of A549 cells by inducing endoplasmic reticulum (ER) stress and mitochondrial dysfunction (39, 40). Patients with low degrees of PCSK9 had an excellent response to ICI therapy, which has enabled the development of PCSK9-based absolute biomarkers or scientific drugs (39-41).

**1.3. PCSK9 and Breast Cancer**

Breast cancer is a common disease in women worldwide, with 1.5 million women diagnosed each year (42). Pseurotin A (PS) is a special spiro heterocyclic γ-lactam alkaloid from the fungal culture of *Pseudeurotium ovalis* (38). Many studies have found that PS inhibits PCSK9 secretion in breast cancer by targeting BALB/c mice (38). While lipids were related to lung and colorectal cancer risks, no association was found between lipids and the histological characteristics of breast cancer tumors (43, 44). But still, in 2008 research by Shah* et al.*, indicates that triglycerides may be negatively related to breast cancer risk, whereas HDL-C might protect postmenopausal women from breast cancer (45, 46). Coexisting physiological factors, such as an underlying metabolic syndrome, post-menopausal state, or chemotherapy, might impact the amounts of circulating lipids, potentially obscuring the relationship between lipid profile and breast cancer prognosis (42-44, 46). As a result of these studies, inhibiting PCSK9 may even improve breast cancer behavior and hold promise as a diagnostic and prognostic biomarker.

**1.4. PCSK9 and Prostate Cancer**

Prostate cancer (PC) is the second most commonly diagnosed cancer in men and the fifth cause of death globally (47, 48). PCSK9 siRNA therapy dramatically improved cell survival, reduced apoptosis, and protected lymph node carcinoma of the prostate (LnCap) against cell damage by increasing the expression of cytochrome C (cyto C), B-cell leukemia/lymphoma 2 (Bcl-2), and Bcl-2 associated X protein (49, 50). According to the convincing evidence derived from large-scale genetic data, the therapeutic suppression of lipid-lowering medications targeting PCSK9 may lessen the incidence of prostate cancer (51). Recent research shows that genetically mediated regulation of PCSK9 is significantly linked to a decreased risk of both overall and early-onset prostate cancer, perhaps through a mechanism involving the reduction of Lp(a) levels (47, 50-52).

**Table 1:** Amount of PCSK9 in different carcinomas

Cancer Name	Most Influential Factor	Expression Level	Refs
Hepatocellular carcinoma	GSTP1, JNK signaling pathway, and HepG2	Upregulated	(1, 27, 30, 38)
Lung Cancer	Adenocarcinoma human alveolar basal epithelial cells (A549)	Downregulated	(21, 40)
Breast Cancer	Pseurotin A (PS) – BALB/c	Upregulated	(38, 44, 46)
Prostate Cancer	Cyto C, Bcl-2, and Bax-LnCap	Downregulated	(32, 47, 50, 51)

**2. PCSK9 and Interaction with MHC-I**

**Fig. 3:**
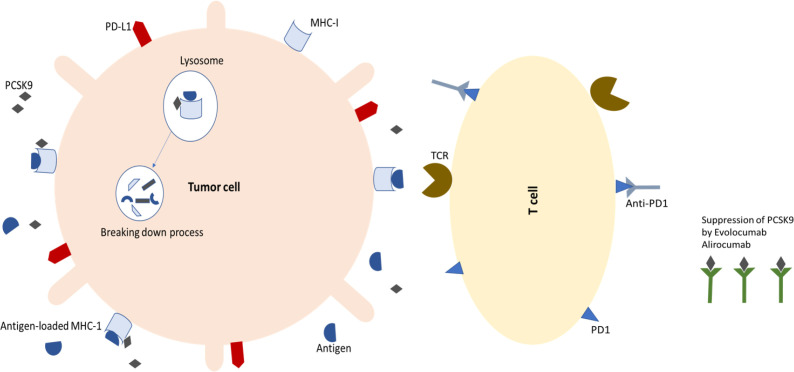
T cells attack tumor cells by binding to antigens on MHC-I molecules on their surfaces, initiating the adaptive immune response. On the other hand, with the connection of PD1 and anti-PD1 antibodies, the possibility of immunosuppression is neutralized. Both of these functions increase the anti-cancer effect. As a result of PCSK9 inhibition, T cells infiltrate the tumor, making it susceptible to immune checkpoint therapy (23, 58).

Major histocompatibility complex 1 (MHC-I) is synthesized by ER, then assembled with beta 2-microglobulin and stored by the ER until loaded with antigenic peptides (53). Within the cytosol, MHC-I molecules attach to the antigens and infectious agents such as viral particles and tumor-derived molecules (54). The expression of MHC-I proteins on cancer cells increases when PCSK9 is inhibited, resulting in a massive influx of cytotoxic T lymphocytes (Figure 1) (23). T Lymphocytes and the effector chemicals are essential for regulating spontaneous, induced, or transplanted immunity (55). Xinjian Liu* et al.*, demonstrated that CD8+, CD4+ T helper cells (Th), T cells, and natural killer cells (NKCs) were significantly increased in PCSK9-deficient tumors in a flow cytometry study (23, 56). According to their findings, PCSK9-deficient tumor cells have many T-cell receptors (TCRs) and a wide diversity of mature T-cells (23). As a result of MHC interaction with TCRs, other stimuli trigger the immune response. T lymphocytes and other immune cells, such as macrophages, eliminate MHC-I positive or heterogeneous tumor cells (5). This is a novel finding regarding how PCSK9 regulates cell surface MHC-I and thus influences intra-tumoral immune infiltration. Thus, it is possible that neutralizing PCSK9 encourages intra-tumoral T-cell infiltration and makes tumors more susceptible to immune checkpoint therapy (Figure 3) (57). 

**3. Response to T-cell, MHC-I, PD-1 & CTLA-4**

The immune response requires two signals to activate T cells; triggered by B7.1/CD80 or B7.2/CD86 interacting with naive T cells and the TCR (59, 60). CTLA-4 inhibits T cell activity by binding to B7 and is being studied as a potential prognosis marker in cancer treatment (15). PD-L1 is a ligand for the immune checkpoint PD-1, and its interaction negatively regulates T cell activation (61, 62). Antibodies targeting PD-1 or PD-L1 have become the new standard cancer treatment (15, 63). Increased CTLA-4 and PD-1/PD-L1 expression is associated with poorer overall survival (62). Tumor-immune evasion commonly involves decreased MHC-I expression and increased immune checkpoint ligands on the cell surface (64, 65). PD-L1 expression in tumors can serve as a biomarker for the treatments inhibiting this molecule, and a higher level of MHC-I expression indicates a potential response to the immune checkpoint therapy (5, 66, 67).

**4. PCSK9 & Immune Checkpoint Therapy**

Now, it is possible to understand how immune checkpoints affect tumor development and how PCSK9 plays a role in this way (68). The immune checkpoints are inhibitory pathways vital for the self-tolerance and maintenance of collateral tissue protection by modulating the immune responses and their length (68). Thus, it is clear that tumors choose immune checkpoint pathways as a primary immune resistance mechanism, especially against T cells specific to the tumor antigens (53); therefore, they may be a potential target for cancer immunotherapy. In immunotherapies, the patient's immune system is used to fight cancer (23). They may overcome resistance mechanisms associated with other medicines by directly targeting the immune system (69). The balance between tumor cells and the immune system allows the tumor to grow uncontrollably and shift in favor of the tumor (46). The emergence of tumor cells with decreased immunogenicity is an example of this escape system, which dampens the anti-tumor immune response for tumor elimination (70). The ICIs have been used for decades to reactivate the immune system by inactivating checkpoint inhibitory proteins on cancer cells or T cells and to help the immune system detect and attack cancer cells (71). PD-1, CTLA-4, lymphocyte activation gene-3 (LAG-3), T cell immunoglobulin and mucin domain-containing protein 3 (TIM3), T cell immunoreceptor with immunoglobulin and ITIM domain (TIGIT), and B- and T-lymphocyte attenuator (BTLA) (Table 2) are few examples of inhibitory immune checkpoint receptors, which were discovered and studied in cancer (60). Treatment of different cancers requires selectively targeting immune checkpoints with specially designed checkpoint-blocking antibodies (like CTLA-4 and PD-1) (63). Thus, PCSK9 inhibition has been proposed as a potential strategy to improve immune checkpoint treatment by inhibiting proteins that suppress checkpoint signaling pathways in T cells and improving their reaction to tumor cells (20).

**Table 2:** Inhibitory Immune checkpoint receptors (ICRs).

ICRs	Expressed on	A mechanism on T cell	Marker For	Ref
*PD-1*	All T cells during activation.	Connection by PD-L1 leads to rapid termination of TCR intracellular signaling and inhibition of T cell proliferation.	- Angioimmunoblastic lymphoma - Downregulates immune responses	(53)
*CTLA-4*	All T cells during activation.	Decreasing the function of T cells.	Downregulates immune responses	(72)
*LAG-3*	Activated T cells, natural killer cells, B cells, and plasmacytoid dendritic cells	Encouraging differentiation into T regulatory cells	Offensive progression in different human tumors such as; - Melanoma, - Hodgkin's lymphoma, - Chronic lymphocytic leukemia, - Colorectal cancer, - Ovarian cancer, etc.	(73)
*TIM-3*	Interferon-γ-producing CD4+ and CD8+ T cells. Monocytes	Suppress T-cell responses upon interaction with their ligand(s).	Activation marker of macrophages and an inhibitor of macrophage activity	(74)
*TIGIT*	Activated T cells are also found in NK cells	Binds to T cell receptors and triggers direct inhibitory signals.	T-Cell lethargy in Liver Cancer	(75)
*BTLA*	CD4/CD8 single-positive T-cells	BTLA inhibits T-cell reactions and cytokine production	Demonstrate putative permissive activation state of B cell subtypes in healthy blood donors.	(76)

## Conclusion

The present study shows PCSK9 functions through many mechanisms, including regulation of several cellular receptors, controlling circulating LDL, and apoptosis pathways, and regulation of the immune response to the tumor cells (71). The discovery that PCSK9 modulates cell surface MHC-I levels and intratumoral immune infiltration is novel in terms of mechanism. When PCSK9 is inhibited, various malignancies respond better to immune checkpoint therapy (64). Furthermore, our findings show that a combination of Alirocumab and Evolocumab significantly may reduce cholesterol levels by inhibiting PCSK9 (32). Based on previous studies, ICIs are strongly associated with activated T cells. Several immune checkpoints, including CTLA-4, PD-1, LAG3, and TIM3, inhibit immune system activity; blocking them triggers immune responses against cancerous cells (60). Finally, more individualized tumor genetics-based immune checkpoint combination approaches (such as PCSK9) must be researched. Despite numerous challenges, there is optimism that checkpoint inhibitors are paving the way to a new cancer treatment era.

## Ethical Approval

This article does not contain any studies with human participants or animals performed by any of the authors.

## Funding

None.

## Conflict of Interest

The authors declared no conflict of interest.
